# Multi-Aperture CMOS Sun Sensor for Microsatellite Attitude Determination

**DOI:** 10.3390/s90604503

**Published:** 2009-06-09

**Authors:** Giancarlo Rufino, Michele Grassi

**Affiliations:** Department of Aerospace Engineering, University of Naples Federico II, 80 Piazzale Tecchio, 80125, Naples, Italy; E-Mail: michele.grassi@unina.it

**Keywords:** sun sensor, satellite attitude determination, calibration, neural networks

## Abstract

This paper describes the high precision digital sun sensor under development at the University of Naples. The sensor determines the sun line orientation in the sensor frame from the measurement of the sun position on the focal plane. It exploits CMOS technology and an original optical head design with multiple apertures. This allows simultaneous multiple acquisitions of the sun as spots on the focal plane. The sensor can be operated either with a fixed or a variable number of sun spots, depending on the required field of view and sun-line measurement precision. Multiple acquisitions are averaged by using techniques which minimize the computational load to extract the sun line orientation with high precision. Accuracy and computational efficiency are also improved thanks to an original design of the calibration function relying on neural networks. Extensive test campaigns are carried out using a laboratory test facility reproducing sun spectrum, apparent size and distance, and variable illumination directions. Test results validate the sensor concept, confirming the precision improvement achievable with multiple apertures, and sensor operation with a variable number of sun spots. Specifically, the sensor provides accuracy and precision in the order of 1 arcmin and 1 arcsec, respectively.

## 1. Introduction

Recent achievements and future trends in microengineering have identified micro/nano-technologies as enabling technologies for future space programs. Indeed, in recent years more and more microsatellites and nanosatellites have been developed. They have much lighter mass, much lower power consumption, cost and smaller size than conventional space platforms, so they are easier to design, manufacture and launch. Moreover, they can be flown in formation so as to synthesize a larger platform. Nevertheless, to keep their capabilities comparable with the ones of larger platforms, it is necessary to scale down components and subsystems. In this context, the development of integrated micro-accelerometers, micro-gyros, and modern CMOS-based electro-optical sensors for highly accurate navigation and attitude determination is one of the most challenging and interesting task, coexisting the demanding requirement of autonomy for aerospace GN&C (Guidance, Navigation and Control) systems. Autonomy of operation can be reliably achieved by fusion of the outputs of more sensors. In this perspective, the integration of micro/nano-technology devices is very promising to obtain compact, reduced mass, low power systems. With the aim of miniaturization of electro-optical sensors for navigation and attitude determination, Active Pixel Sensor (APS) technology is playing a dominant role since it offers the possibility of integrating detector and electronics into a single chip. The latest generation of star trackers and sun sensors adopting such components are currently being studied and developed [1,2].

Sun sensors are essential components of a satellite, since they can provide coarse-to-medium accuracy measurements of the sun's direction in the satellite-fixed axes, which is an essential part of the information needed for autonomous attitude determination in the various phases of a space mission. Conventional sun sensors are too large and costly to be used on board micro- and nanosatellites. Hence, in recent years, a new generation of sun sensors has emerged which relies on imaging devices. These sensors adopts linear or planar CCD or APS as focal plane detectors and a mask placed on the top at a certain distance. The mask has tiny slits or pinhole apertures to produce sun images on the focal plane from which the sun line direction can be extracted. These sensors offer medium-to-high measurement accuracy, depending on the optical head design and the algorithms used to process sun images [3-7]. In this context, the research team at the University of Naples is developing a two-axis digital Micro Sun Sensor (MSS) based on an APS photodetector. The sensor is being developed as part of a program sponsored by the Italian Space Agency (ASI) to fly a number of innovative technology payloads on board the first Italian microsatellite platform, MIOsat, scheduled for flight in 2011 [8].

The sensor provides the sun line orientation in the sensor-fixed reference frame by measuring the sun image position on the focal plane produced by a mask with one or more tiny apertures. High accuracy in the sun line determination is achieved thanks to an original design of the calibration function based on neural networks, which allows it to overcome the limitations of using calibration procedures based on geometrical models only. High precision is achieved by exploiting an original design of the optical head in which an array of tiny apertures is created on the mask. Indeed, this particular design provides multiple simultaneous images of the sun disk on the focal plane, and, than multiple measurements of the sun line orientation, which can be averaged to filter out random noise components. In this case, a specific solution is envisaged to construct the calibration function in order to limit the computational load, thus allowing sensor operation in real-time. The multi-aperture configuration improves precision with respect to classical configurations based on one single aperture [4-7], but, of course, restricts the measurable range of sun line orientations of an amount depending on the number of exploited sun spots as in [3]. To overcome this limitation, a major innovation is introduced, which consists in operating the sensor also in an extended mode, i.e. by processing a variable number of spots, at the cost of reducing precision for increasing off-boresight angles. As it will be shown in the paper, this mode allows us to double the angular range of sensor operability. Since ASI requires using COTS components as far as possible for the flight unit development, the sensor model used for on-ground concept validation and testing relies on commercial components. Specifically, the sensor model presented in the paper is the evolution of a previous prototype [9-11] in which both optical head and processing unit have been upgraded in view of the flight unit development, based on COTS components. Sensor testing was executed using a ground facility in which in-orbit illumination conditions are reproduced as closely as possible (e.g. sun spectrum and apparent angular diameter) and several parameters can be remotely controlled (e.g. sensor exposition time, illumination direction) during the experiments. As described in the paper, the main goals of the indoor test campaign are validating the innovative sensor design, by verifying precision improvement achieved with the multi-aperture design with respect to the single-aperture solution, testing performance of the neural calibration procedure, and validating sensor operation in extended mode. Description of the envisaged algorithms and calibration procedures, and discussion of the results achieved in two test campaigns, in which the sensor is operated both in basic (i.e. with a fixed number of sun spots) and extended (i.e. with a variable number of sun spots) modes are the main focus of the paper.

The paper is organized as follows: the sensor concept and operating principle are described in Section 2, the sensor hardware and software prototypes are presented in Section 3, the test facility is described in details in Section 4, and, finally, Section 5 reports test campaigns and results.

## 2. Sensor Concept and Operating Principle

**Figure 1.**
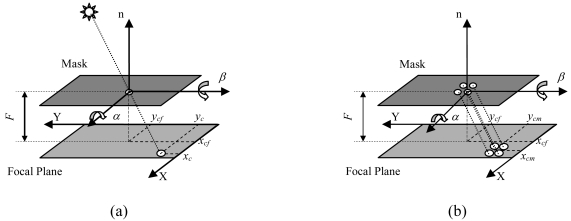
Sensor operating principle with one-hole mask (a) and multi-hole mask (b).

The basic functionality of the sensor is the computation of the instantaneous position of the sun in the sensor reference frame, i.e., the orientation of the illumination direction in the sensor FOV (Field of View). This can be accomplished by imaging the sun and then reconstructing the sun-line unit vector components on the basis of the location of this image on the sensor Focal Plane (FP) (see Figure 1a).

This sensor scheme envisages the following components:

– an image forming system, to observe the sun;
– a focal plane equipped with a two-dimensional photodetector, to acquire the formed sun image;
– a computing unit (CPU), to carry out processing of the acquired images and generate the desired measures.

The components of the first and second items have been conceived as a single functional unit, that will be referred to as Optical Head (OH) in the following. It is worth noting that the scene to be imaged by the OH has peculiar characteristics: the subject of interest is the sun, that is extremely bright, and the background is mostly dark in unperturbed conditions because it contains stars, that are much less bright and tinier light sources. Also, the processing of the acquired images, as discussed in the following, does not need sharp images as input. Hence, a simple pinhole-like aperture is typically adopted for the image forming system [3,7]. The latter one is the natural evolution of slit apertures in use with former systems based on one-dimensional detectors [12,13]. Adoption of such a lens-less solution results into a great benefit for sensor reliability, compactness, cost, and robustness, that are of great interest, especially for a space system. Thus, the image forming system can be reduced to an opaque mask with a tiny hole through which the incoming light reaches the detector sensing surface and forms an image of the sun as a bright spot. Shape and size of the spot reproduce those of the mask hole. The size of the latter one, the detector sensing surface area, and its distance from the mask, that can be regarded as the system focal length (*F*), determine the sensor FOV.

The sensor developed by the authors presents a major innovation in the image forming system design, which is composed of a mask with multiple tiny holes arranged in an array. In this case, a bright spot is imaged for each hole of the mask, and all the spots cover almost the same area as the holes of the mask. This allows improved precision in sun-line determination. In fact, multiple images of the sun can be simultaneously produced on the focal plane (see Figure 1b) [9,14]. and exploited to generate multiple, simultaneous measures of the instantaneous sun-line, one for each image. Then, an improved estimate of the sun-line direction can be computed by averaging them. This results into improved precision of measurements since random noise components, that are present in each single-image-based estimate, are filtered out. In principle, assuming uncorrelated noise contributions, precision improvement is related to the square root of the number of measures used in the averaging operation as:

(1) $$\sigma_{N}=\frac{\sigma_{1}}{\sqrt{N}}$$

where σ_1_ is the uncertainty in one single measure.

Thanks to this original solution, precision performance comparable to those of modern star trackers can be achieved, as it will be shown in the following sections of the paper. However, minor drawbacks exist: additional computational load to manage multi-spot processing, and significant reduction of the sensor FOV consequent to the larger area of the sensing surface occupied by the imaged spots. Both these aspects are addressed in the following, where effective solutions are proposed. Finally, the introduction of a multi-hole mask allows operating the sensor also with a variable number of spots, thus getting flexibility on FOV, precision and rapidity of response in the sun line determination. Hence, the same sensor could be used in different phases of the missions in which different requirements, in terms of sensor FOV, precision and rapidity of response, apply (e.g. coarse and high precision modes).

## 3. Sensor Prototype

A laboratory model of the digital sun sensor implementing the enhanced, multi-hole configuration has been realized by the authors with the aim of characterizing the enhanced configuration and assessing its performance with respect to the basic one (i.e. the one-hole configuration), developing and testing algorithms for system operation, operating and testing COTS solutions for the MSS flight unit in view of the MIOsat mission.

### 3.1. Hardware Model

**Figure 2.**
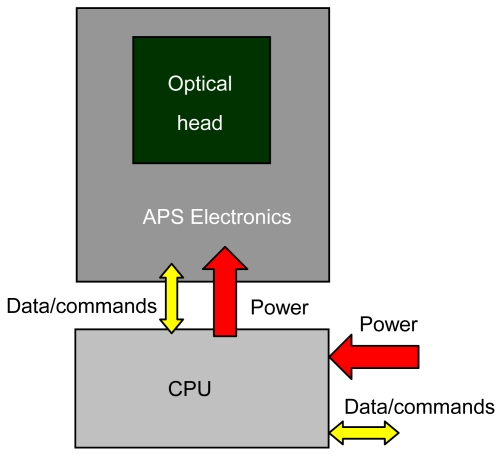
Sensor architecture.

A sensor architecture consisting of two distinct hardware units (see Figure 2), corresponding to its two functional units, OH and CPU, is selected for the model to get on-board installation flexibility for the flight validation.

Based on the objectives of the MSS experiment within MIOsat program, sensor components are defined by selecting COTS parts for both the OH and the CPU. Parts not available on the market, like the mask, are designed and realized specifically, making use of standard materials and manufacturing processes as far as possible. It is worth noting that the current sensor model, which is the evolution of a previous prototype [9-11], is intended mainly as a proof of concept, i.e. it is developed with the main intent of testing on ground, and, then, in flight, innovative concepts and solutions for the optical head, as well as calibration and processing algorithms. For this reason, the sensor design it is not optimized in terms of miniaturization of some of its components.

The OH includes the CMOS photodetector, the focal plane electronics and the mask. The photodetector is a two-dimensional CMOS Active Pixel Sensor (APS) produced by Micron Technology Inc.™. This unit includes a 10-bit ADC, programmable electronics for some basic camera functions (i.e. shutter time setting, windowing, row/column skipping), and part of the focal plane electronics. Focal plane electronics and I/O interface to the CPU are implemented in two commercial boards, also by Micron. The board containing the detector implements all the needed focal plane circuitry for detector control and image acquisition, whilst the other board operates conversion of the detector specific communication protocol to the USB 2.0 standard.

As previously specified, the opaque mask was designed in house by the authors. It is manufactured out of a very thin, plain steel foil, and the required holes are realized by electron discharge, a low-cost, micro-manufacturing process that allows precision up to 0.01 mm in hole size and shape. It is worth noting that the use of such a metal foil for the mask, being its thickness comparable to the hole size, determines that imaged spots are distorted as the sun-line gets far from the sensor boresight. However, it represents a systematic effect that can be completely compensated for, at least theoretically, by the Calibration Function (CF). Nevertheless, for increasing off-boresight angles, summed to the reduced irradiance toward the focal plane, it reduces the acquired image quality. For the flight unit, an aperture protection filter will be added to the mask, so that the solar radiation reaching the focal plane will not saturate the photodetector also thanks to proper shutter time setting. Filter and shutter time will be tuned to keep sun-spot pixel signals within 70 % of full scale, and to allow for the desired output rate. By doing so, when celestial bodies other than the sun are in the sensor FOV during real operation, their radiation reaching the focal plane will only generate signals within the image noise already present, being the sun much brighter (e.g., > 420,000 times than full moon and 30,000 times than earth albedo).

**Figure 3.**
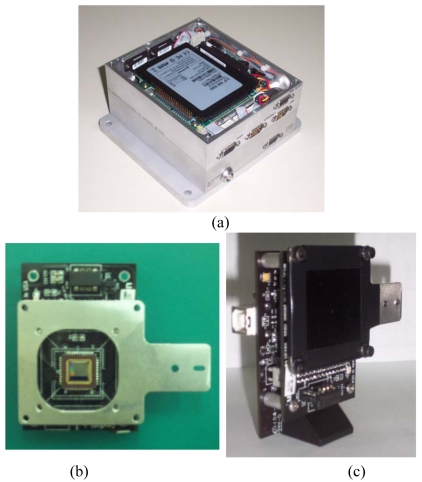
Preliminary design of the UniNa MSS for MIOsat mission: CPU (a) and OH focal plane electronics without and with the mask (b and c, respectively) installed on the interface for installation in the test facility.

To test both basic and enhanced sensor configurations, two different masks are realized, with one and 100 holes, respectively, arranged in a 10 × 10 array. Hole size is the same (0.1-mm radius) in both cases, which determines imaged spots with diameter of about 60 pixels for the adopted detector. A mechanical interface (mask holder, see Figure 3) is designed and realized ad hoc to couple the focal plane to the mask, in accordance with the nominal design configuration, in which mask and detector sensing surface are parallel, at a distance equal to the focal length, and their centers are aligned so that the resulting FOV is symmetrical with respect to the boresight axis. The mask adapter also includes a support for the sensor installation in the test facility. Figure 3 shows pictures of the two sensor functional units. Specifically, Figure 3(b) shows the sensor board and the mask holder adopted for sensor testing in the laboratory test facility. Table 1 reports the OH nominal technical characteristics.

The CPU is a single-board computer in pc-104 format by RTD™. It is a COTS product designed for operation in harsh environment (i.e. extended temperature, conduction-based cooling). It includes all the needed peripherals and interface in a single board, and it is also equipped with a pc-104 power-conditioning module to regulate the power input from the unregulated bus and to supply both the CPU and the OH. The latter one, in particular, is powered by the CPU at 5 Vdc via the USB link, which is also used for CPU-OH data exchange.

A solid state mass memory is adopted to get reliability of operation in space. Accurate analyses are being carried out for both the CPU and the OH electronics to assess critical technologies for space operations, so to identify viable solutions to guarantee the reliability and lifetime needed for the experiment execution and goals. In this context, typical space environment problems (i.e. radiation, vacuum, thermal) are being addressed. In particular, space radiation shielding adequate for a two-years lifetime will be guaranteed by specifically designed, aluminum enclosures (see Figure 3). Preliminary mass and power budgets of the sensor model are reported in Table 2. It is worth noting again that mass and power consumption values refer to the commercial units adopted for the sensor prototype development to allow proof of concept on ground and in flight.

### 3.2. Software Model

It includes the routines for sensor operation management, image acquisitions, I/O to the satellite OBDH, and the routines implementing the algorithms for sun line determination. These algorithms, which are the topic of the next sub-sections, basically consist of image processing algorithms, to evaluate the FP position of the sun image(s), and calibration algorithms, which use position information to extract the sun line orientation:

Image processing algorithm basically perform image preprocessing, such as noise reduction, and computation of the FP position of the spot-like sun image(s). Specifically, once the spot has been localized to pixel accuracy, its sub-pixel position is computed as the centroid of the multi-pixel image as [15]:

(2) $$\begin{array}{cc} {\text{x}}_{\text{c}}=\frac{\sum_{\text{k}=1}^{\text{n}}{\text{x}}_{\text{k}}{\text{I}}_{\text{k}}}{\sum_{\text{k}=1}^{\text{n}}{\text{I}}_{\text{k}}}, & {\text{y}}_{\text{c}}=\frac{\sum_{\text{k}=1}^{\text{n}}{\text{y}}_{\text{k}}{\text{I}}_{\text{k}}}{\sum_{\text{k}=1}^{\text{n}}{\text{I}}_{\text{k}}} \end{array}$$

where x_c_ and y_c_ are the centroid FP co-ordinates, x_k_ and y_k_ are the FP co-ordinates and I_k_ is the intensity of the generic pixel in the considered n-pixel integration window (including the whole spot). Of course, centroiding of Equation (2) is applied after some image pre-processing, that is noise removal, spot search and rough localization necessary to select the contriding integration window. Details about the adopted algorithms and the error sources affecting centroid determination can be found in [9-11].

The above processing does not need any particular specialization when applied to the multi-spot configuration. Indeed, in this case an array of spots must be imaged and processed. Preliminarily, the whole array is localized on the FP and its size is checked out to verify if it is only partly imaged due to a large off-boresight of the sun or any acquisition problem. Then, a centroid should be computed for each of the spots to produce multiple estimates of the same illumination direction, which should be then averaged to get highly precise sun line determination. Nevertheless, such a strategy would require the management of a large number of sun-line computations. Indeed, a dedicated CF would be needed for each spot of the array, i.e., a dedicated function should be constructed after calibration tests, implemented in the on-board software, and run at the time of sensor operation. The latter two aspects are particularly critical, since they impact flight-unit design and operation leading to more demanding requirements. To overcome these critical aspects of the enhanced configuration operation, the averaging is carried out at the stage of centroid computation. Hence, a centroid is computed for each imaged sun spot and, then, they are averaged to extract an average centroid. The latter one is dealt with as the single-spot centroid of the basic sensor configuration. In this way, the additional computational load required by the multi-hole configuration is limited to the computation of multiple centroids.

The CF maps the sun spot computed FP position into the sun line orientation, which is described by the two angles α and β in Figure 1. They represent the sequence of rotations needed to align the sensor boresight axis with the illumination direction.

The authors have considered and tested several solutions to implement the mapping function [11], aiming at achieving computational efficiency in terms of simplicity, by requiring neither numerous parameters nor complex computations, and accuracy over the whole sensor FOV. Very simple schemes based on the following basic geometrical model:

(3) $$\begin{array}{c} x=-F\tan\beta +x_{cf}\\ y=-F\frac{\tan\alpha }{\cos\beta }+y_{cf} \end{array}$$

were tried, showing poor accuracy, especially for large sun-line off-boresight angles. Indeed, the geometrical model in Equation (3) does not take account of unavoidable misalignments of sensor components and, more in general, deviations from the design nominal configuration. In addition, only scarce improvements are achieved by using more complex geometrical models in which additional parameters are introduced to model the actual mask-FP geometry (i.e. focal length and component misalignments) estimated during sensor calibration by LSQ best fit. In fact, additional non-linear effects are present, due to mask thickness, manufacturing tolerances, diffraction effects, non-uniform response and finite size of photodetector pixels, which cannot be explicitly included in the model. Hence, in spite of the poor results, there would be a significant increase in the complexity of the model which impacts negatively both the calibration procedure and sensor operation.

Neural-network-based CFs provide a viable and interesting alternative solution to achieve high accuracy without introducing very complex models. Indeed, Neural Networks (NNs) with supervised training are universal approximators, i.e., they can approximate to any desired degree of accuracy any real-valued, continuous function (or *sufficiently regular* function, with a countable number of discontinuities between two compact sets). Moreover, NNs, if non-linear w.r.t. their parameters, are also parsimonious, that is they implement the desired approximation using the lowest number of parameters [16,17]. Hence, they can be effectively exploited to build the CF, since they can implement the required mapping without any prior assumption about the centroid-to-sun-line transformation, by constructing the non-linear mapping on the basis of experimental data only.

**Figure 4.**
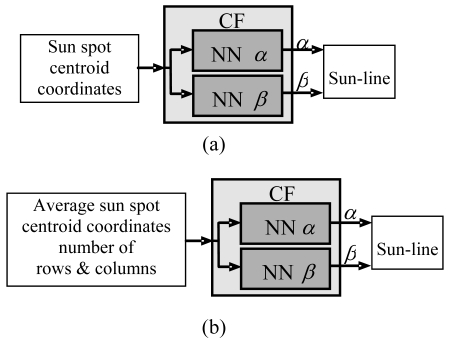
Implemented NN-based calibration function: Standard FOV (a) and Extended FOV (b).

Multilayer feed-forward NNs with sigmoid activation function in the hidden layer and linear output neurons are considered for this application. In particular, the selected NN structure consists of one hidden layer. Different NN architectures characterized by a different number of neurons in the hidden layer have been tested. Two distinct NNs are used to compute α and β independently. Several solutions (see Table 3) in terms of NN input/output variables were compared in previous, dedicated test campaigns [11]. Specifically, fully-neural CFs were built and compared to mixed models in which neural corrections were introduced to refine results of the geometrical models. It was found out that the most satisfactory trade-off is represented by the scheme in Figure 4(a), which shows the lighter computational load and very good accuracy on a wide FOV. It is worth mentioning that the number of neurons in the hidden layer cannot be uniquely fixed, but it is peculiar to each case and has to be determined during calibration.

The FOV size in the basic operation mode, referred to as Standard FOV (S-FOV) in the following, is reduced during operation with multiple sun spots by the requirement that all the imaged spots lie within the detector sensing area. Hence, for a given detector, using a large number of sun images improves sensor precision at the cost of reducing the measurable range of off-boresight illumination directions. The theoretical FOV size of the UniNa MSS prototype is in the order of 40 × 20 [10] for the mask with a 10 × 10-array of holes. It is worth noting that size and aspect ratio of the sensor FOV are determined by the sizes of the detector sensing area and of the imaged array of spots. Hence, even for a given detector, FOV size and aspect ratio can be customized by modifying the array of holes on the mask, in terms of number, size and arrangement of the holes.

It is possible to widen the range of measurable illumination directions by accepting that a reduced number of spots lies within the detector sensing area (eXtended FOV, X-FOV). By doing so, the computation of the sun-line is based on a variable number of spots. In particular, the larger the off-boresight angle is, the lower the number of usable spots gets. This fact determines a reduced precision at the FOV edges because of the lower number of simultaneous measurements that can be averaged to produce the final result. Also a new calibration function must be implemented, valid over the whole useful X-FOV and capable of accounting for the variable number of exploited sun spots. Of course this calibration function should not introduce any performance loss at the X-FOV center.

**Figure 5.**
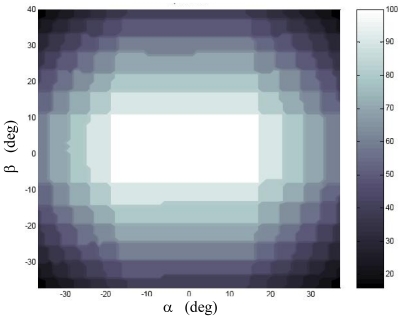
Number of sun spots imaged by the UniNa MSS at variable off-boresight illumination directions (experimental data).

The above described X-FOV mode is implemented in the sensor prototype and tested on ground. To this aim, also an enhanced centroiding algorithm is developed. It computes the average centroid of a set of sun spots arranged in a two-dimensional array, assuming that the number of rows and columns can be variable. For the calibration function, the same neural structure of the S-FOV mode was maintained. However, the NN input stage is modified so to exploit also the imaged spot array size (i.e. row and columns) to map the average centroid into the illumination direction (see Figure 4b). In the X-FOV operating mode, the sensor gets a theoretical FOV larger than 80 × 70 [18]. Figure 5 shows the number of correctly imaged spots in the extended FOV, as obtained in laboratory tests.

## 4. Laboratory Test Facility

A dedicated laboratory facility has been designed and realized to carry out tests and calibration of wide-FOV sun sensors. Basically, its functionality consists in illuminating the sensor under test from an accurately known direction. The facility is designed to simulate sun illumination in earth orbit: light source appearing as at approximately 1 AU, angular size of 0.53 deg, and spectral distribution reproducing the solar one. Sun radiation intensity is not simulated, being this aspect reproducible by modifying the sensor shutter time and/or the sensor entrance aperture protection filter [11]. The facility allows reproducing a variable illumination direction which can be known with high accuracy. Attention is paid to the reproduction of sun radiating flux characteristics, i.e., spectral composition and density, since they influence sensor detector response and, hence, sensor output.

**Figure 6.**
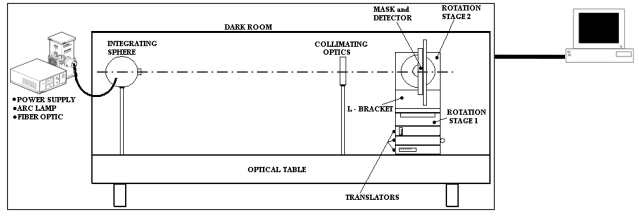
Laboratory Test Facility schematic.

The test system consists of four main sections: radiation source, collimating optics, sensor micro-positioning subsystem, PC-based control terminal [10,11]. They also allow sensor-light source relative pointing control. These components are installed on an optical table for stable and precise alignment. A dark room set-up covers all the components to isolate them from external light sources and avoid internal reflections (see Figure 6).

### 4.1. Radiation Source Subsystem

A 1,000-W Xenon arc-lamp by Oriel Instruments™ is used as radiation source. In fact, the spectrum of emission of this kind of lamp allows them to be used in sun simulators when the spectral region from 200 nm to 2,500 nm is of interest [19]. The latter is wider than the one necessary for the present application, that is determined by the detector spectral response band (400 – 1,000 nm). The lamp is powered by a stabilized 70 Vdc supplier with controllable output in the range 450 – 1,000 W. The housing where the lamp is installed is equipped with a water filter to cut off infrared emission beyond 1,200 nm, and optics focusing the light into a 48 mm-diameter collimated beam. This output is conveyed by means of a special adapter and a fiber optics to a 10-cm-diameter integrating sphere by Labsphere™. Its internal surface, covered with Spectraflect™ coating, guarantees diffuse reflection and low loss (reflection coefficient equal to 0.977 @ 600 nm [20]). At its output port a diffuse and highly uniform emission is available, as desired. It supplies a light source adequate to illuminate the sensor under test. Table 4 summarizes subsystem characteristics.

### 4.2. Collimating Optics

Under real operating conditions, the radiation illuminating the sensor is practically collimated as a result of the large distance from the sun. Of course, the angular spread of light rays consequent to the finite angular size of the sun is present. This is reproduced by installing a collimating optics before the sensor at a distance from the sphere output port equal to its focal length. The latter is chosen equal to 1.5 m to satisfy size constraints of the facility. Consequently, a 13.7 mm diameter circular aperture is installed at the output port to obtain the desired angular size (0.53°) of the uniform radiating source. In this configuration, the radiant flux density of the sun is not reproduced because it would require very high power levels and high hardware complexity. On the other hand, satisfactory test can be carried out by increasing the sensor shutter time so that the selected average pixel output at the spot centre is obtained. This value is set to 80 % of the linear response limit of the pixel. Of course, this approach leads to conservative results in terms of sensor measurement accuracy because increased noise levels are present due to dark current effects for longer exposure time.

### 4.3. Sensor Micro-positioning Subsystem

This allows accommodating the sensor in the test camera and rotating it with respect to the light source. It is designed to allow one to control the sensor boresight and to modify it by means of rotations along two mutually perpendicular axes, that are also perpendicular to the longitudinal axis of the test system. Relative motion is implemented by means of three high-precision micro-translators and two rotation stages by Physik Instrumente™. The subsystem is based on a three-dimensional micro-translator which is introduced to operate the fine alignment of the sensor boresight to source and collimator axes. It is realized by stacking a vertical translator and two horizontal ones. They can be adjusted manually and guarantee 2.5 μm of accuracy [21]. The two rotation stages, whose main features are in Table 5, are moved by servomotors and controlled via a PC serial link. The first rotation stage is installed on the top of the three-dimensional translator. It operates rotations along the first axis. A L-shaped aluminum bracket is specifically designed to assemble the second rotation stage in order to operate rotation around the second axis. The bracket is designed in conjunction with the mask holder so that, nominally, the two rotation axes and the sensor boresight are mutually perpendicular and intersect at the mask centre. As a result, the rotation axes and the sensor boresight axis (n) form the sensor-fixed reference frame when the sensor is installed in the test camera.

## 5. Test Campaigns

Laboratory tests are carried out with two major objectives: validating the sensor concept and assessing the achievable performance improvement with the multi-hole mask, and validating original solutions such as neural CF and X-FOV operating mode. Specifically, two different test campaigns are executed: one aiming at evaluating and comparing performance of the multi-hole configuration with the basic one (i.e. the 1-hole mask), the other to evaluate performance in the X-FOV mode, when images with a variable number of spots are acquired. To this end, the first test campaign is performed on a restricted portion of the FOV (about [-20°:20°] × [-10°:10°]) in which 100 spots are always imaged (see Figure 5); whilst the second test campaign covers a much wider FOV (about [-40°:40°] × [-35°:35°]), in which images with different numbers of spots are acquired (with a minimum of 16 spots as a 4 × 4 array) depending on the sun line orientation. This allows significantly extending sensor operability range.

During these tests, the prototype OH and CPU are operated in the laboratory facility to acquire sun images which are then processed in off-line mode on the control workstation by running the described algorithms as Matlab™ codes. During the tests the MSS CPU controls OH operation and stores the acquired images based on commands from the control workstation.

To the aim of a thorough comprehension of the presented test results, it is useful to describe the adopted test procedure in details. Specifically, in both the test campaigns the following steps are performed:

1. preliminarily, three distinct sets of orientations are defined, each uniformly sampling the relevant FOV. Two sets, referred to as Training Set (TrS) and Training Test Set (TTS), are dedicated to NNs training, the third one, the Test Set (TS), to sensor performance evaluation;
2. as second step, the sensor is operated in the laboratory facility. By keeping the illumination direction unchanged, a fixed number of images is acquired. This is repeated for each of the orientations in TrS, TTS, and TS. The number of acquisitions is selected so to be adequate for meaningful statistical analysis of the computed parameters at each illumination direction [22];
3. as third step, all images are processed off-line to compute the centroids of imaged spots and the average centroid;
4. based on TrS and TTS data, two NNs are trained for each configuration of the sensor, as described in the following;
5. then, the sun line is measured through the NN-based CF for each acquisition at the TS orientations;
6. finally, performance is evaluated by comparing the measured sun lines to the their TS known values. Errors are computed as the difference between the computed angles defining the sun-line orientation and the known angles of the sun simulator facility, accurately known thanks to the rotation stage feedback to the control workstation. Also, the angle between the computed sun line and the known one, referred to as sun line error in the following, is computed. It is a useful, synthetic figure of merit, being fully representative of measure performance.

Prototype performance are evaluated in terms of accuracy and precision as defined in [23]. Specifically, the following parameters (referred to as local performance parameters) are computed at each FOV orientation and regarded as performance figures of merit:

– mean of the errors of the computed angles and sun-lines (local accuracy);
– maximum of the errors of the computed angle and sun-lines (worst-case local error);
– standard deviation of the errors of the computed angle and sun lines (local precision).

Mean and standard deviation of local performance parameters are computed over the whole FOV (hence they are referred to as global performance parameters) to estimate average sensor performance and relevant dispersion in the whole FOV, respectively.

### 5.1. S-FOV Test Campaign

**Figure 7.**
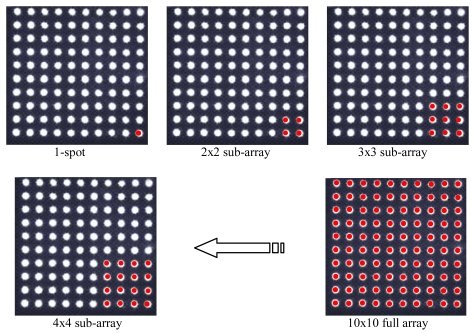
Spot sub-array sequence for multi-hole configuration testing.

Objective of the first test campaign is the validation of the enhanced sensor concept, and the assessment of its performance with respect to the basic sensor configuration. Various multi-hole configurations (2 × 2, 3 × 3, 4 × 4, and 10 × 10 arrays) are considered for a thorough characterization of the enhanced sensor concept. To avoid construction of several mask prototypes, multiple acquisition campaigns, and generation of an enormous bulk of data, all the considered multi-hole configurations are evaluated by using the same data set acquired with the 100-hole mask, including the 1-hole case. In fact, fixed sub-arrays of the imaged spots in all the acquisitions of the campaign are selected according to the sequence of Figure 7 and dealt with as acquired alone. This procedure avoids comparing results of different campaigns, which could introduce undesired effects due to different operating conditions.

However, interference from spots which are not used for sun-line determination is certainly present and not accounted for, but, reasonably, it does not impact the main goal of the test campaign which is evaluating sensor precision improvement with increasing spot number. Data relevant to the selected sub-arrays are processed independently from calibration, i.e., NNs training, to performance assessment.

**Figure 8.**
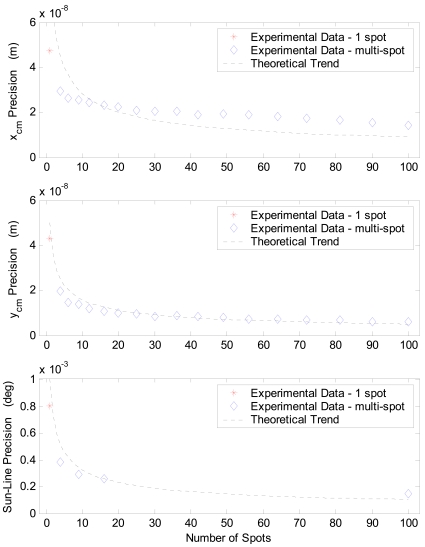
Sensor precision performance in S-FOV operating mode for different numbers of sun spot exploited to compute the average centroid.

With the aim of thorough sensor precision characterization, in addition to the above multi-spot configurations other cases are considered by selecting extra, intermediate array size (also rectangular ones) within the 10 × 10 available full array. However, in these cases the processing is limited to the average centroid computation. This is sufficient to analyze precision as a function of the number of acquired spots. Indeed, sun-line measure fluctuations, i.e. local precision, directly derives from average centroid fluctuations for a given illumination direction. Hence the mentioned precision analysis is carried out in terms of average centroid for a much larger number of cases. Results of this test campaign are shown in Table 6 and in Figure 8. Specifically, in Table 6 results are organized so to show sensor average performance and performance uniformity over the S-FOV. In particular, average performance is evaluated by averaging over the FOV local accuracy, worst-case local error and local precision; whilst performance uniformity is computed as dispersion (i.e. standard deviation) over the FOV of local accuracy, worst-case local error, and local precision.

The beneficial effect of having multiple apertures, with respect to the case of one single aperture, can be clearly identified. Indeed, average precision is significantly improved (from about 0.001° to about 0.0002°); whilst, average accuracy, which is in the order of 0.01°, is not influenced. To highlight this effect, in Figure 8 average precision in terms of centroid and sun-line error is reported as a function of the number of simultaneously imaged spots and compared to the expected trend for uncorrelated, multiple measurements (Equation 1). A limited number of experimental data is reported for the sun line error due to the reduced number of calibration functions that were trained. As expected, a certain degree of correlation exists, which is more marked in x_cm_ and in the sun line error. Hence, the achieved improvement, although fairly evident, is lower than the theoretical one.

### 5.2. X-Fov Test Campaign

**Figure 9.**
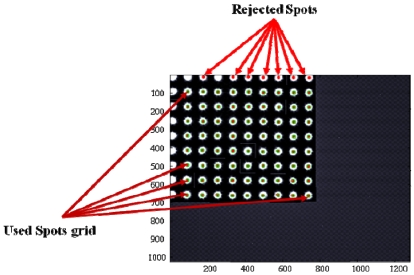
Example of imaged spot rejection in X-FOV operating mode when observing the sun at large off-boresight angles.

The second test campaign is focused on testing the X-FOV operating mode. Main objectives are validating the neural calibration procedure using the spot array size and the average centroid as additional inputs, and assessing the achievable performance over the wider FOV allowed by this solution. In this case, different TrS, TTS, and TS are defined, extending over a wider range of illumination directions with respect to the sensor S-FOV. Acquisition with at least 16 correctly imaged sun spots are considered. Figure 9 shows an example of an off-boresight image acquisition in which one row and one column of spots are not correctly imaged and, hence, they are rejected before running algorithms for computing spot centroids and their average.

Table 7 reports the same global statistics as Table 6 but relevant to X-FOV mode performance. To perform comparative analyses, statistics are evaluated both over the whole X-FOV and over its S-FOV restriction. Average precision over the X-FOV, which is in the order of 0.0003°, is comparable to that of the S-FOV case, with little worsening imputable to the reduced number of correctly imaged spots.

On the other hand, when considering the average precision over the S-FOV restriction, results are practically identical to those of the S-FOV operating mode. Slight discrepancies are due to the selection of different orientation samples to train NNs and evaluate performance.

**Figure 10.**
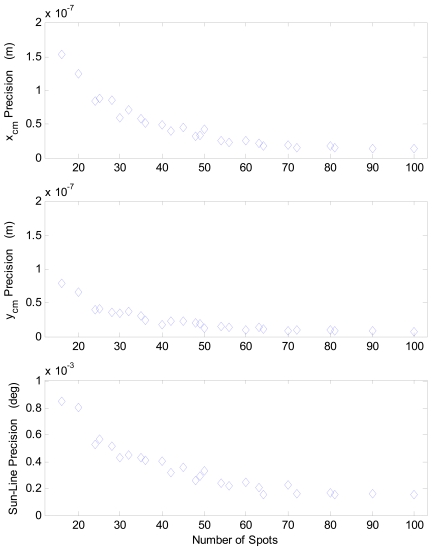
Sensor precision performance in X-FOV operating mode as a function of the number of imaged spots.

**Figure 11.**
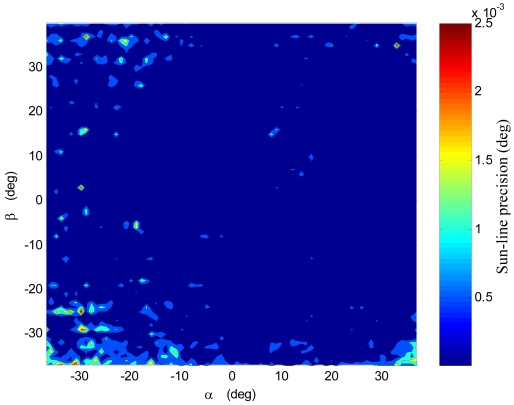
Local Sun-line precision distribution over the extended FOV.

More in-depth analyses are reported in Figure 10, which shows the average precision as a function of the number of sun spots exploited in the computation of the average centroid. The same trend as for the S-FOV test campaign is recognized, in agreement with the expected theoretical behavior. Regarding Figure 10, it must be considered that the number of cases, on which statistics for different number of used spots are based, is not constant but as resulting from uniformly sampling the X-FOV (see Figure 5). Finally, Figure 11 plots the precision distribution over the X-FOV, showing good uniformity in area around the boresight. Performance degrades in the regions where less spots can be exploited, as expected.

Average accuracy, which is in the order of 0.06° for the whole X-FOV and 0.03° in the S-FOV restriction, is worse w.r.t the S-FOV operating mode. This is due to the fact that, in the X-FOV mode, the CF maps a much larger input subset and, hence, two considerations stand:

– the X-FOV global performance is relevant also to larger off-boresight angles, for which performance is worse;
– the NN is trained over the whole X-FOV, so it is less specialized than the previous NN to the S-FOV restriction.

## 6. Conclusions

In this paper a novel sun sensor under development at the University of Naples has been described. The sensor relies on CMOS technology and on an innovative design of the optical head which allows increasing precision in the determination of the sun illumination direction by exploiting a mask with up to 100 apertures. In this way, many simultaneous images of the sun disk can be acquired on the focal plane and, then, averaged to extract the sun line with high precision.

The paper has described the sensor laboratory model which is being developed using COTS components within a project funded by the Italian Space Agency for flying innovative technologies and experimental payloads on board the first Italian microsatellite MIOsat, scheduled for flight by the end of 2011. The paper main focus has been on the original solutions exploited for the development of computationally efficient sensor calibration functions, which are based on neural network, and on the analysis of the results of test campaigns carried out with the sensor laboratory model and test facility to validate the sensor concept and the innovative design of the optical head. Specifically, results of two independent test campaigns have been reported in which the sensor is operated both with a fixed number and a variable number of sun spots using the same neural calibration procedure. Indeed, the second operating mode allows overcoming the unavoidable reduction in the measurable range of illumination directions caused by the operation with 100 sun images. By operating with 100 sun images, which corresponds to [-20°:20°] × [-10°:10°] FOV, sensor average accuracy is better than 0.01°, and average precision improves of almost one order of magnitude with respect to the operation with only one sun image, i.e. from 0.001° to about 0.0002°, very close to the precision of a modern star sensor. Operation with a variable number of sun spots (up to a minimum of 16) allows significantly widening the sensor FOV, up to about [-40°:40°] × [-35°:35°], while preserving average precision performance which only slightly degrades to about 0.0003°. On the other hand, average accuracy reduction is more pronounced (in the order of 0.06°) mainly due to the fact that performance degrades for increasing off-boresight of the sun line. Future work will concern the development and test of the sensor flight unit in view of its flight validation in 2011.

## Figures and Tables

**Table 1.** Main characteristics of the MSS prototype optical head.

Focal length	3 mm
Field of view	94 × 81 deg (basic conf., 1 spot)
	52 × 28 deg (enhanced configuration, 10 × 10 spots)
	90 × 74 deg (enhanced configuration, 3 × 3 spots)

**Mask**	

Material	steel
Thickness	0.1 mm
Number of holes	1/100 (enhanced configuration)
Hole diameter	0.2 mm
Hole arrangement (enhanced configuration)	10 × 10 array, 0.42 mm pitch (both directions)

**Photodetector**	

Technology	CMOS Active Pixel Sensor
Model	MT 9M001 by Micron Technology, Inc.
Resolution	1280 × 1024 pixels
Pixel size	5.2 × 5.2 μm
Sensing area size	6.66 × 5.32 mm

**Table 2.** Preliminary mass and power budget of the MSS sensor prototype.

**MSS Unit**	**Component**	**Mass (g)**	**Power consumption Typ./max (W)**

**CPU**	Single board computer	130	5.6
	SS HD	30	0.06 / 0.12
	Power conditioning unit	170	Efficiency 72 %
	Cooling (heat-pipe based)	40	-
	Case (4-mm Al shield)	1,490	-
	Harness, etc.	140	-

	Total	2,000	< 8.0

**OH**	Sensor board (incl. detector)	35	< 0.4
	Demo board	40	
	Case (4-mm Al shield)	210	-
	Harness, etc.	30	-

	Total	315	< 0.4 (via USB i/f to CPU)

**CPU & OH**	Total	2,315	< 8.4

**Table 3.** Input and output of the NN-based solutions for the sensor calibration function tested in [11]. The best-trade-off solution is in italics.

**Input**	**Output**

*Spot centroid coordinates*	*α angle*
	*β angle*
α and β as computed by model (3) and nominal sensor parameters	α angle
	β angle
α and β as computed by model (3) and LSQ estimate of sensor parameters	α angle
	β angle

**Table 4.** Main characteristics of the light source subsystem in the laboratory facility for sun sensor test and calibration.

**Arc-lamp (Oriel Instrum.™)**		**Integrating sphere (Labsphere™)**		**Collimator (Oriel Instrum.™)**	

Power	1,000 W	Sphere diameter	101.6 mm	Diameter	50.8 mm
Light flux	30,000 Lumens	Input port diameter	25.4 mm	Focal length	1,500 mm (589 nm)
Life time	1,000 Hrs	Output port diameter	13.7 mm	Refractive index	1.5167 (589 nm)
Bulb diameter	38 mm	Internal reflectance	0.97772	Centre thickness	6.4 mm

**Table 5.** Main features of the rotation stages of the micro-positioning subsystem in the laboratory facility for sun sensor test and calibration.

Rotation range	Continuous
Design resolution	0.001 deg
Min. incremental motion	0.001 deg
Max. velocity	90 deg/s
Unidirectional repeatability	0.00342 deg
Wobble	0.00860 deg
Encoder resolution	4,000 counts/rev
Motor power / voltage range	30 W / 0-24 Vdc
Weight	0.62 kg

**Table 6.** S-FOV test campaign results: comparison of enhanced configurations (multi-hole mask) to the basic sensor (1-hole mask).

**Number of Spots**	**α(deg)**					
	**β(deg)**					
	**Sun-line error(deg)**					

	**Average Performance over S-FOV**			**Performance Dispersion over S-FOV**		
	*Accuracy*	*Worst-case error*	*Precision*	*Accuracy*	*Worst-case error*	*Precision*

**1 of 100^-^**	0.0103	0.0114	7.17 × 10^-4^	0.0085	0.0075	8.65 × 10^-4^
	0.0097	0.0107	8.09 × 10^-4^	0.0114	0.0089	8.52 × 10^-4^
	0.0157	0.0168	8.03 × 10^-4^	0.0091	0.0091	9.96 × 10^-4^

**4 of 100**	0.0096	0.0101	3.04 × 10^-4^	0.0056	0.0046	2.81 × 10^-4^
	0.0125	0.0131	4.19 × 10^-4^	0.0123	0.0097	3.11 × 10^-4^
	0.0170	0.0175	3.82 × 10^-4^	0.0082	0.0082	1.89 × 10^-4^

**9 of 100**	0.0093	0.0096	2.27 × 10^-4^	0.0049	0.004	1.96 × 10^-4^
	0.0130	0.0134	3.15 × 10^-4^	0.0136	0.010	1.89 × 10^-4^
	0.0171	0.0175	2.93 × 10^-4^	0.0083	0.0083	1.89 × 10^-4^

**16 of 100**	0.0094	0.0096	1.71 × 10^-4^	0.0047	0.0038	1.49 × 10^-4^
	0.0130	0.0134	2.88 × 10^-4^	0.0136	0.0105	1.51 × 10^-4^
	0.0172	0.0176	2.57 × 10^-4^	0.0085	0.0085	1.78 × 10^-4^
**100**	0.0094	0.0095	8.43 × 10^-5^	0.0049	0.0035	6.58 × 10^-5^
	0.0137	0.0139	1.85 × 10^-4^	0.0148	0.0099	8.86 × 10^-5^
	0.0176	0.0178	1.47 × 10^-4^	0.0077	0.0080	8.11 × 10^-5^

**Table 7.** X-FOV test campaign results: average performance of the enhanced sensor over the eXtended FOV (variable number of viewed spots) and over the S-FOV (100 viewed spots).

**α(deg)**					
**β(deg)**					
**Sun-line error(deg)**					

**Average Performance over X-FOV**			**Performance Dispersion over X-FOV**		
*Accuracy*	*Worst-case error*	*Precision*	*Accuracy*	*Worst-case error*	*Precision*

0.0375	0.0377	1.61 × 10^-4^	0.0505	0.0340	6.79 × 10^-4^
0.0443	0.0447	3.38 × 10^-4^	0.0576	0.0372	4.22 × 10^-4^
0.0625	0.0628	2.51 × 10^-4^	0.0393	0.0394	6.84 × 10^-4^

**Average Performance over S-FOV**			**Performance Dispersion over S-FOV**		
*Accuracy*	*Worst-case error*	*Precision*	*Accuracy*	*Worst-case error*	*Precision*

0.0198	0.0199	9.10 × 10^-5^	0.0236	0.0179	7.11 × 10^-5^
0.0187	0.0189	1.98 × 10^-4^	0.0235	0.0146	1.01 × 10^-4^
0.0305	0.0307	1.47 × 10^-4^	0.0180	0.0180	9.57 × 10^-5^
